# The effect of temperature, gradient, and load carriage on oxygen consumption, posture, and gait characteristics

**DOI:** 10.1007/s00421-016-3531-7

**Published:** 2017-02-02

**Authors:** Katrina Hinde, Ray Lloyd, Chris Low, Carlton Cooke

**Affiliations:** 1 Leeds Beckett University, Headingley Campus, Leeds, West Yorkshire LS6 3QS UK; 2 Leeds Trinity University, Brownberrie Lane, Horsforth, Leeds, LS18 5HD UK

**Keywords:** Load carriage, Cold exposure, Oxygen consumption, Stride length

## Abstract

**Purpose:**

The purpose of this experiment was to evaluate the effect of load carriage in a range of temperatures to establish the interaction between cold exposure, the magnitude of change from unloaded to loaded walking and gradient.

**Methods:**

Eleven participants (19–27 years) provided written informed consent before performing six randomly ordered walking trials in six temperatures (20, 10, 5, 0, −5, and −10 °C). Trials involved two unloaded walking bouts before and after loaded walking (18.2 kg) at 4 km · h^−1^, on 0 and 10% gradients in 4 min bouts.

**Results:**

The change in absolute oxygen consumption (V̇O_2_) from the first unloaded bout to loaded walking was similar across all six temperatures. When repeating the second unloaded bout, V̇O_2_ at both −5 and −10 °C was greater compared to the first. At −10 °C, V̇O_2_ was increased from 1.60 ± 0.30 to 1.89 ± 0.51 L · min^−1^. Regardless of temperature, gradient had a greater effect on V̇O_2_ and heart rate (HR) than backpack load. HR was unaffected by temperature. Stride length (SL) decreased with decreasing temperature, but trunk forward lean was greater during cold exposure.

**Conclusion:**

Decreased ambient temperature did not influence the magnitude of change in V̇O_2_ from unloaded to loaded walking. However, in cold temperatures, V̇O_2_ was significantly higher than in warm conditions. The increased V̇O_2_ in colder temperatures at the same exercise intensity is predicted to ultimately lead to earlier onset of fatigue and cessation of exercise. These results highlight the need to consider both appropriate clothing and fitness during cold exposure.

## Introduction

Recreational trekking and mountaineering is becoming increasingly popular and affordable and activities, such as these, alongside numerous occupational tasks, usually require external loads to be carried. Loads can be carried in various ways, but in mountaineering, trekking and military operations, the backpack is recommended as the most versatile, appropriate, and economical method of manual load carriage (Knapik et al. 1996). The relationship between load carried and energy expenditure has been extensively researched. The general consensus regarding energy expenditure when carrying loads on the back is that as load increases, oxygen consumption and thus energy expenditure increase in an approximately proportional manner (Quesada et al. 2000; Lloyd and Cooke 2000; Faghy and Brown 2014). However, new evidence suggests that during very heavy load carriage (up to 45 kg), the increases in oxygen consumption (V̇O_2_) are not proportional to the load mass (Phillips et al. 2016a). The energy cost of carrying such loads not only depends on the weight of the load, but also varies depending on the age, ethnicity, body mass of the participant, speed, terrain, and biomechanical factors (Givoni and Goldman 1971; Hainsman 1988; Phillips et al. 2016b).

It has been widely reported that V̇O_2_ increases during submaximal exercise in the cold compared to a moderate ambient temperature (Sandsund et al. 1998; Oksa et al. 2004; Jett et al. 2006). Doubt (1991) concluded that there were two possible reasons for higher exercise V̇O_2_ in the cold. The first is non-exercising thermogenesis (shivering), and in the absence of shivering, an elevated V̇O_2_ suggests non-shivering thermogenesis (NST). NST, a facultative form of thermogenesis, includes muscle tensing, feeling of stiffness and enhanced metabolism (Parsons 2003) being closely linked with brown adipose tissue (BAT) activity (Nedergaard et al. 2007). It is unknown at what environmental temperatures, NST becomes active. The second mechanism, not independent from the first, for higher exercise V̇O_2_ in the cold is the potential decrease in mechanical efficiency (Doubt 1991; Oksa et al. 2004).

There is considerable literature surrounding load carriage, but most of this work is laboratory based in thermo-neutral environments. There is little known about the interactive effects of cold exposure and load carriage which is surprising given both factors are commonly experienced together. The literature surrounding cold exposure mainly focuses on cycling performance and a comparison between two temperatures (warm and cold), although some studies compare three temperatures. There have only been two other investigations using a range of six ambient temperatures (Sandsund et al. 2012; Renberg et al. 2014), but these were concerned with running performance, employing higher exercise intensities (67–100% V̇O_2max_) with participants wearing adequate winter (ski) clothing. This is the first investigation into the effects of a range of decreasing temperatures on physiological variables during walking. In addition, there are very few studies assessing the effect of temperature on biomechanical variables, in particular, stride parameters. Limited evidence suggests that cold exposure elicits an increase in stride frequency and a decrease in stride length (SL, Folland et al. 2006). Furthermore, there are equivocal findings associated with stride parameters and walking on a gradient. Much of the existing literature involves studies that focus solely on the biomechanical or physiological effects of load carriage, with very few taking a combined factor approach (Simpson et al. 2011), ignoring the reality that such effects can interact.

The purpose of the present study was to investigate the effect of load carriage in a range of temperatures to establish the interaction between cold exposure and the magnitude of change from unloaded to loaded walking when studying V̇O_2_, heart rate (HR) and skin temperature. In addition, the interaction between gradient and load was evaluated to establish which stressor has a greater effect on oxygen consumption and whether the combined effect is equal to, less than or greater than the individual stressors.

We hypothesised that oxygen consumption would increase as temperature decreased and thus the change in V̇O_2_ when going from unloaded to loaded walking would be greater during cold exposure. Skin temperature and, therefore, HR would be significantly reduced during cold exposure. It was also hypothesised that a combination of increased gradient, load and decreased temperature would all interact to create a greater associated oxygen consumption and increases in SL.

## Methods

### Participants

University students (*n* = 11; seven males, four females) volunteered to participate in the study (age, 22 ± 3 years; height, 173.9 ± 8.6 cm; body mass, 71.31 ± 10.44 kg). The experimental procedures were approved by the Local Research Ethics Committee of the Carnegie Faculty at Leeds Beckett University. Prior to testing, participants were all informed of the procedures and potential risks of the experiments. All participants gave written informed consent.

Participants were apparently healthy, aged 18+ years and assessed as “low-risk” individuals by the ACSM (2013) Guidelines for cardiovascular, pulmonary, and metabolic diseases. If after answering questions on risk factors, signs, symptoms, and history of disease, participants had no risk factors or a maximum of one then they were classed as low risk. Areas assessed for risk factors were age, family history, smoking status, lifestyle, obesity, and hypertension. Participants were habitually active, completing 3–5 sessions of moderate exercise a week and were able to easily tolerate 2 h of submaximal exercise relative to their fitness levels.

### Exercise protocol

A short exercise protocol of ~50 min (involving 24 min of walking) was completed in an environmental chamber (TISS, Peak Performance Chamber Series 2009, Hampshire, UK) at Leeds Beckett University. Environmental conditions were kept constant and controlled at 50 ± 5% humidity, ~160 mmHg partial pressure of oxygen and with a wind speed of 2.9 m · s^−1^. Participants completed six trials, one at each temperature (20 ± 0.5, 10 ± 0.5, 5 ± 0.5, 0 ± 0.5, −5 ± 0.5, and − 10 ± 1.0 °C). Participants’ exposure to the different environmental temperatures was assigned using a Counter-balanced Latin square and at least 24 h separated trials. Participants wore shorts, t-shirt, and trainers for all trials, and for trials at 0 °C and below, hat and gloves were worn.

Before any exercise took place, participant’s resting blood pressure and resting heart rate were measured to ensure that they were apparently healthy and safe to test. Resting blood pressure was measured using the Boso Medicus (Bosch, Jungingen, Germany) manual blood pressure device, whilst resting heart rate was measured using a Polar T31 coded™ transmitter and FT1 watch (Polar, Kempele, Finland). Values for blood pressure of <140/90 mmHg were accepted as normal, as were resting HR values of <100 beats · min^−1^. Baseline skin temperature was also measured using a Squirrel Data Logger (400 Series: 401/451, Wessex Power, Dorset, UK). Skin temperature was measured at four sites (chest, forearm, thigh, and calf), and mean skin temperature (MST) was calculated using the equation created by Ramanathan (1964): MST = [0.3 (chest + arm)] + [0.2 (thigh + leg)].

The protocol began with a 15-min standardised rest period during which heart rate and skin temperature were measured. Core temperature during −10 °C exposure was measured using an ingestible CorTemp® pill (HQInc, Palmetto, Florida) for safety reasons and to ensure that core temperature did not drop below 35.5 °C. Participants then performed unloaded walking on a calibrated treadmill (PPS-med 1, Woodway, Weil am Rhein, Germany) at 4 km · h^−1^ for 4 min bouts at 0 and 10% gradient. 1 min was given after walking at 0% for the treadmill to increase to 10% grade, during which the participant was stationary. Following a rest period allowing for heart rate to return to resting level (7.05 ± 1.55 min), the same walking trial was completed; however, this time participants were loaded, carrying an 18.2 kg backpack. Another rest period was set (6.31 ± 1.58 min) followed by the unloaded section being repeated again. An exercise duration of 4 min was chosen as previous studies by Lloyd and Cooke (2000) have shown that 3 min of exercise is sufficient for participants to be walking to achieve steady-state oxygen consumption. The authors can confirm that steady-state V̇O_2_ was achieved and there were no significant differences between the third and last minute V̇O_2_ readings for any of the environmental conditions.

Heart rate (HR) was monitored continuously throughout the protocol and averaged over the last minute of each stage. Expired gas was analysed by an online gas analyser (Cortex 3B Metalyzer, Leipzig, Germany). Data were collected breath-by-breath, but evaluated over 30 s intervals. Reported V̇O_2_ data were calculated from the last minute of each stage. Participants wore a facemask attached to the sample line which was connected to the online gas analyser. Calibration of the online system was performed before each trial, and values were accepted if the offset was within 0.02% of the actual concentrations.

Participants were filmed from the side using a standard video camera (Casio EX-FH100, New Jersey, USA) set at 60 Hz. The camera was set up 2.58 ± 0.03 m from the treadmill and perpendicular to the treadmill belt. A calibration frame was set up and recorded before each participant began a trial. Biomechanical data were collected in the last minute of each exercise bout for 10 s. Comparable to Lloyd and Cooke (2011), three of the events that occur during a single gait cycle were chosen for analysis: heel strike, mid support, and toe-off. Data were analysed using SIMI Motion 8.5.6 (Unterschleissheim, Germany). Stride length (SL) was calculated using the video recordings according to Lloyd and Cooke (2011). A stride was defined as the period between consecutive toe-off events of the left foot. The time taken for six complete strides (12 steps) was measured and then divided by 6 to get cycle time in seconds (Levine et al. 2012). SL was calculated by multiplying the known treadmill speed (1.1 m · s^−1^) by cycle time. Two landmarks (left hip and left shoulder) were identified using markers. Trunk forward lean included three measurements during a randomly selected, complete gait cycle within the 10 s filming period: left heel strike, mid support, and toe-off. An angle of greater than 90° indicated trunk forward lean and angles less than 90° showed backwards inclination.

### Study design

#### Walking speed

A walking speed of 4 km · h^−1^ was selected due to the consistency of its use within load carriage research and its economy in normal adult walking compared to higher or lower speeds (Grenier et al. 2011).

#### Temperature

A control temperature of 20 °C was selected as the highest temperature as it is often reported within the literature (Oksa et al. 2004). The majority of studies employed cycling as their exercise modality and usually only focused on one cold temperature against a control. The selection of −10 °C as the lowest temperature was based on a balance of considerations of commonly experienced temperatures in popular trekking/mountaineering areas (OnTop 2014), a range of temperatures used in previous studies investigating cold air exposure (Timmons et al. 1985; Oksa et al. 2004) and tolerance and discomfort of participants for 1 h wearing minimal clothing.

#### Load

The load carried was made up of items usually taken on trekking/mountaineering trips. One backpack (Wynnster Equador) was used for the whole study and for all participants. The backpack had a 65 L capacity and was fitted to each participant before their trial started. The adjustable ladder on the main section of the backpack was altered depending on participants’ height and participants were encouraged to carry the majority of the weight on their hips rather than the shoulders. The weight of the loaded pack was 18.2 kg with load justification coming from a combination of previous research methodologies (Simpson et al. 2011).

### Statistical analysis

Data were analysed using IBM SPSS 22, with significance tested at 95% confidence intervals (CI), *p* < 0.05. Descriptive statistics (mean ± SD) were calculated for all outcome measures. All data were tested for normality (Shapiro–Wilk), and all data were normally distributed (*p* > 0.05). To assess for differences between conditions, three-way repeated measures analysis of variance (ANOVA) (6 × 3 × 2; temperature × load × gradient) was conducted to establish any significant main effects and interactions, accounting for variability across the whole study. This analysis was followed up with three-way repeated measures ANOVAs (6 × 2 × 2; temperature × load × gradient) which separated the loading conditions to allow the separate research questions to be addressed (i.e., unloaded 1, loaded; unloaded 1 and unloaded 2; unloaded 2, loaded). Post-hoc tests for significant main effects were conducted using a Bonferroni adjustment. Change in V̇O_2_ (L · min^−1^) from unloaded to loaded walking (ΔV̇O_2_) was analysed using a two-way repeated measures ANOVA (6 × 2; temperature × gradient). Mean skin temperature was analysed in a similar way (6 × 6, temperature × time). In cases when the assumption of sphericity was violated, the Huynh–Feldt correction was used. Effect sizes for repeated measures ANOVA were calculated using partial eta squared (*η*_p_^2^). Two-way and three-way interactions were reported, and classification of interactions were identified according to Lloyd and Havenith (2016).

## Results

### Oxygen consumption

#### Effect of individual stressors

**Fig. 1:**
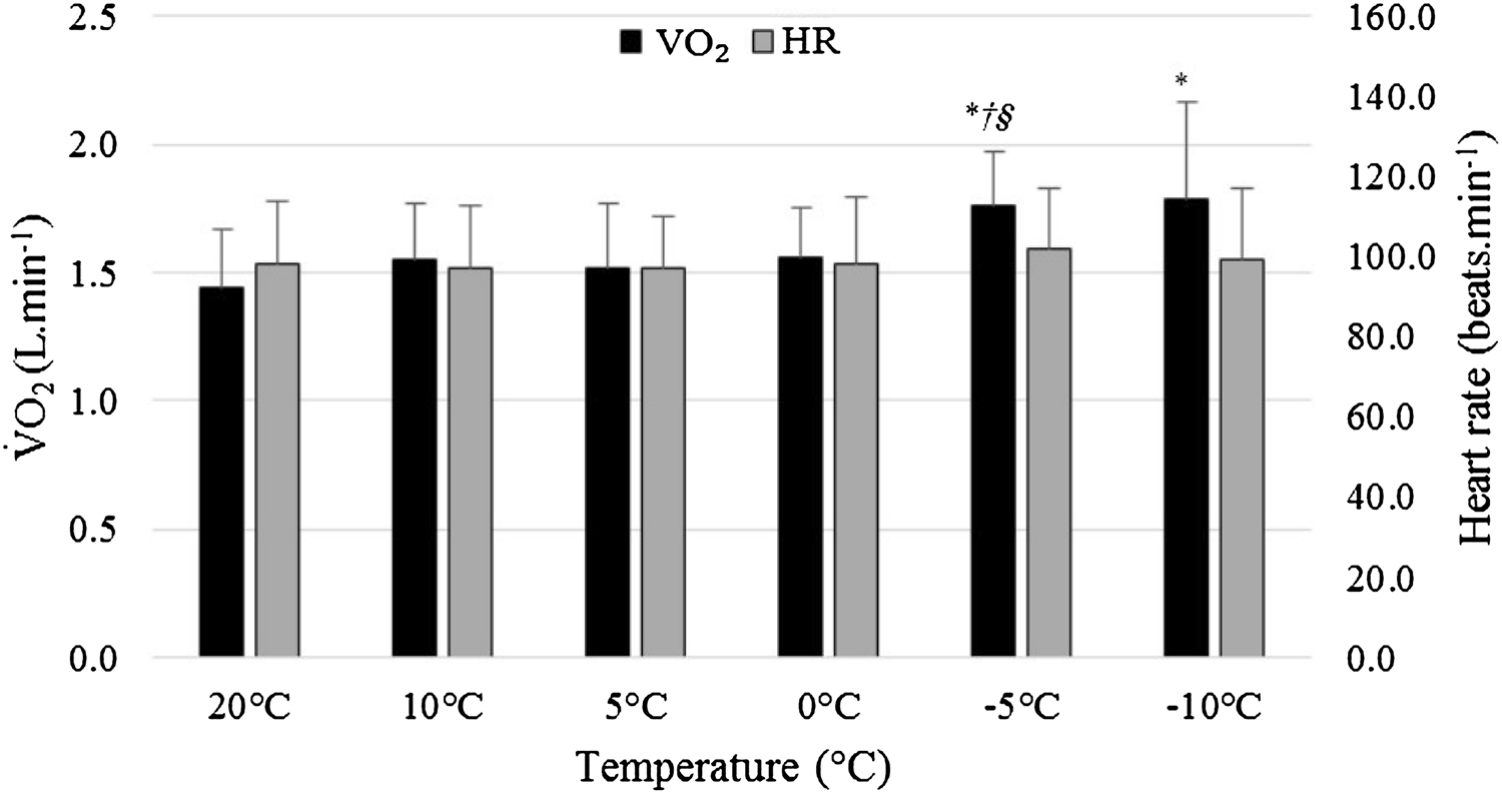
Mean + SD V̇O_2_ values (L · min^−1^) and HR (beats · min^−1^) for the different ambient temperatures averaged across all gradients and loads, *asterisk* denotes a significant difference to 20 °C, *dagger* denotes a significant difference to 10 °C, and *section symbol* denotes a significant difference to 0 °C

Variation in oxygen consumption across all experimental conditions is shown in Table 1. As ambient temperature decreased, V̇O_2_ significantly increased (*p* < 0.001, *η*_p_^2^ = 0.460). Post-hoc tests identified a number of significant differences between ambient temperatures averaged across all loads and gradients (*p* ≤ 0.045, Fig. 1).

**Table 1:** Mean ± SD V̇O_2_ (L · min^−1^) values for each temperature, load, and gradien

	Unloaded 1		Loaded		Unloaded 2	
	0%	10%	0%	10%	0%	10%
20 °C	0.97 ± 0.18	1.65 ± 0.30^e^	1.14 ± 0.18^d^	1.99 ± 0.29^de^	0.95 ± 0.17	1.65 ± 0.28^e^
10 °C	1.03 ± 0.15	1.78 ± 0.28^e^	1.23 ± 0.21^d^	2.17 ± 0.30^de^	1.01 ± 0.13	1.78 ± 0.28^e^
5 °C	1.05 ± 0.19	1.70 ± 0.27^e^	1.24 ± 0.25^d^	2.09 ± 0.37^de^	1.10 ± 0.21	1.74 ± 0.31^e^
0 °C	1.05 ± 0.20	1.74 ± 0.24^e^	1.30 ± 0.16^d^	2.14 ± 0.28^de^	1.25 ± 0.38	1.80 ± 0.29^e^
−5 °C	1.24 ± 0.16^abc^	2.00 ± 0.32^abce^	1.47 ± 0.27^abcd^	2.34 ± 0.29^abcde^	1.56 ± 0.36^abc^	2.23 ± 0.32^abce^
−10 °C	1.21 ± 0.21^a^	1.99 ± 0.46^ae^	1.61 ± 0.40^ad^	2.35 ± 0.59^ade^	1.59 ± 0.42^a^	2.20 ± 0.62^ae^

^a^Denotes a significant difference to 20 °C responses

^b^Denotes a significant difference to 10 °C responses

^c^Denotes a significant difference to 0 °C responses

^d^Denotes a significant difference to unloaded responses

^e^Denotes a significant difference to 0% responses

The repeated unloaded bout of exercise was included to assess variation in low intensity walking and the potential effect of fatigue. When analysing both unloaded conditions, there was a significant main effect for load (*p* = 0.028, *η*_p_^2^ = 0.397), with unloaded 2 producing significantly higher V̇O_2_ values than unloaded 1.

The increase in V̇O_2_ from unloaded to loaded walking did not change significantly with temperature (ΔV̇O_2_, *p* = 0.44, *η*_p_^2^ = 0.089). Although V̇O_2_ was significantly higher when walking uphill (10%), the changes in V̇O_2_ from 0 to 10% gradient were similar across all temperatures (*p* = 0.814, *η*_p_^2^ = 0.043).

#### Differential effects

**Fig. 2:**
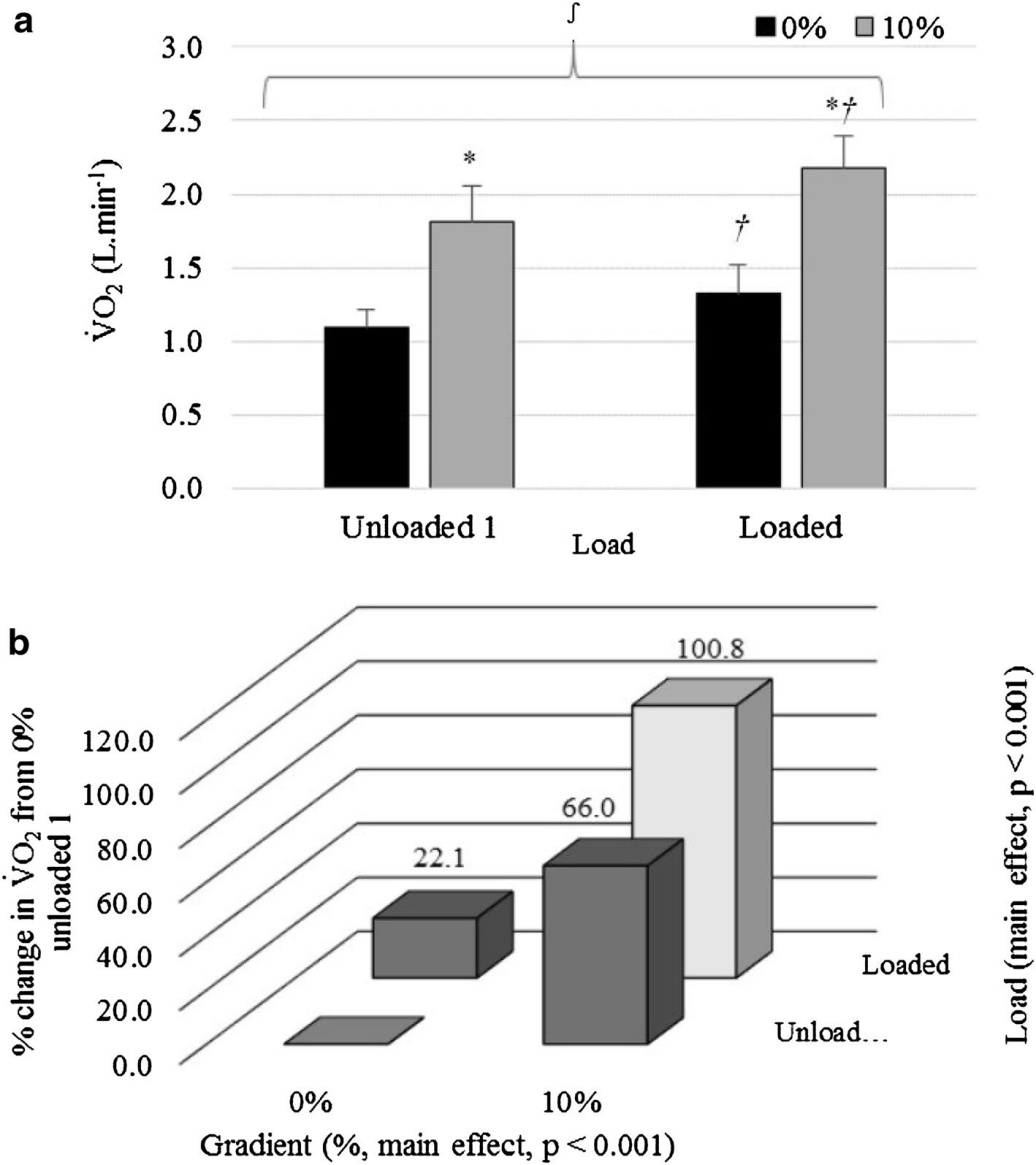
Mean ± SD V̇O_2_ (L · min^−1^) responses for Unloaded 1 and Loaded at 0 and 10% gradient averaged across all ambient temperatures. *Asterisk* denotes a significant difference to 0% responses for the same load, *dagger* denotes a significant difference to unloaded responses for the same gradient, and *integral symbol* denotes an interaction effect for gradient × load. **b** Interaction showing mean percentage change in V̇O_2_ (L · min^−1^) from 0% gradient, unloaded 1, averaged across all ambient temperatures

Further analysis comparing unloaded 1 and loaded conditions showed a significant interaction between load and gradient (*p* = 0.013, *η*_p_^2^ = 0.475, Fig. 2a). Figure 2a shows that gradient had a greater impact than backpack load on V̇O_2_. The effect of load was greater at 10% gradient (mean difference = 0.37 L · min^−1^) than when compared to the change at 0% (mean difference = 0.24 L · min^−1^). In addition, the effect of gradient was greater when participants were loaded than when unloaded (mean difference = 0.85 and 0.72 L · min^−1^, respectively). This interaction can be categorised as a synergistic increase (hyper-additive, Fig. 1b).

**Fig. 3:**
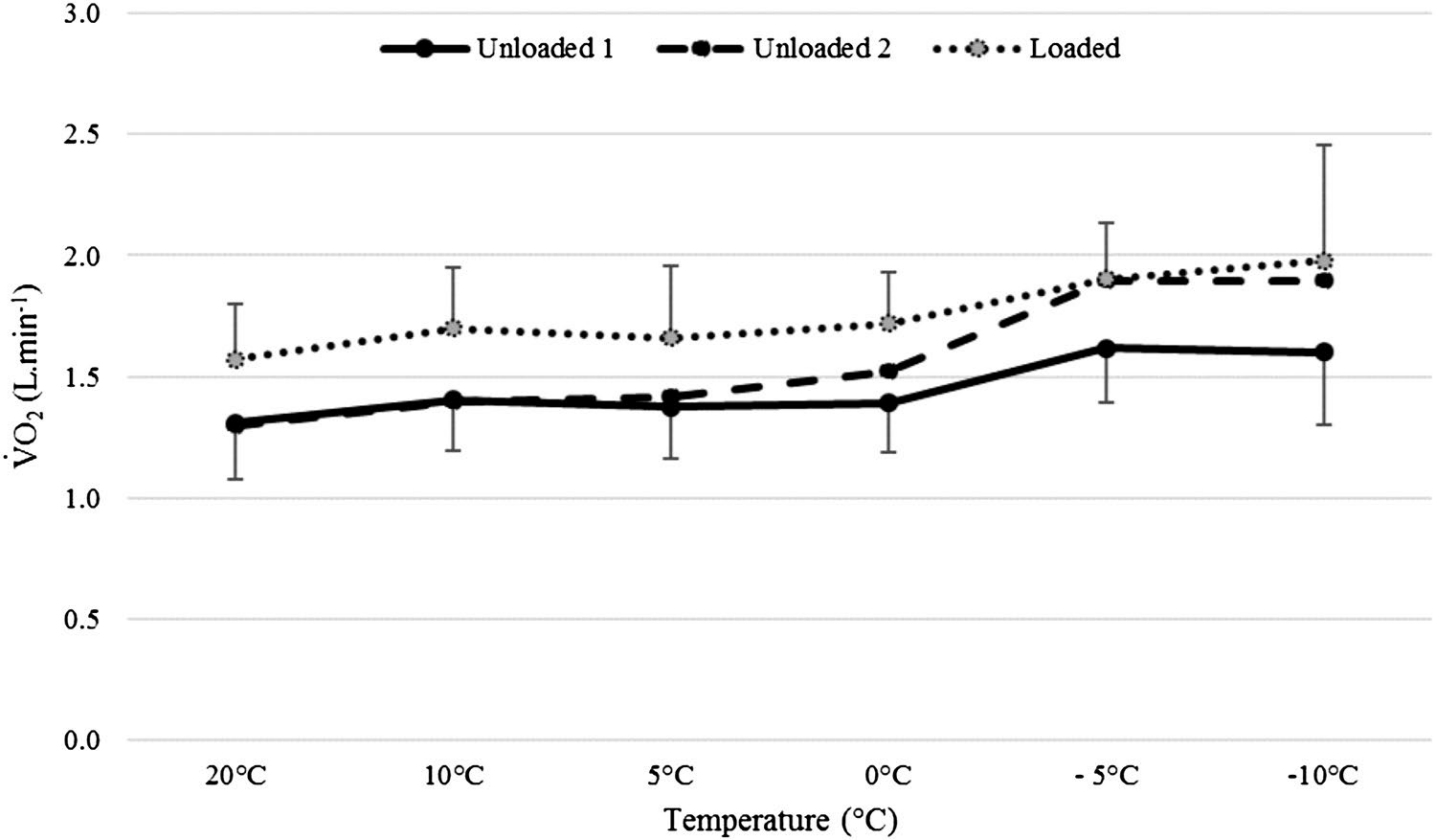
Mean ± SD V̇O_2_ (L · min^−1^) response for Unloaded 1, Loaded and Unloaded 2 phases for all ambient temperatures, averaged across all gradients

**Fig. 4:**
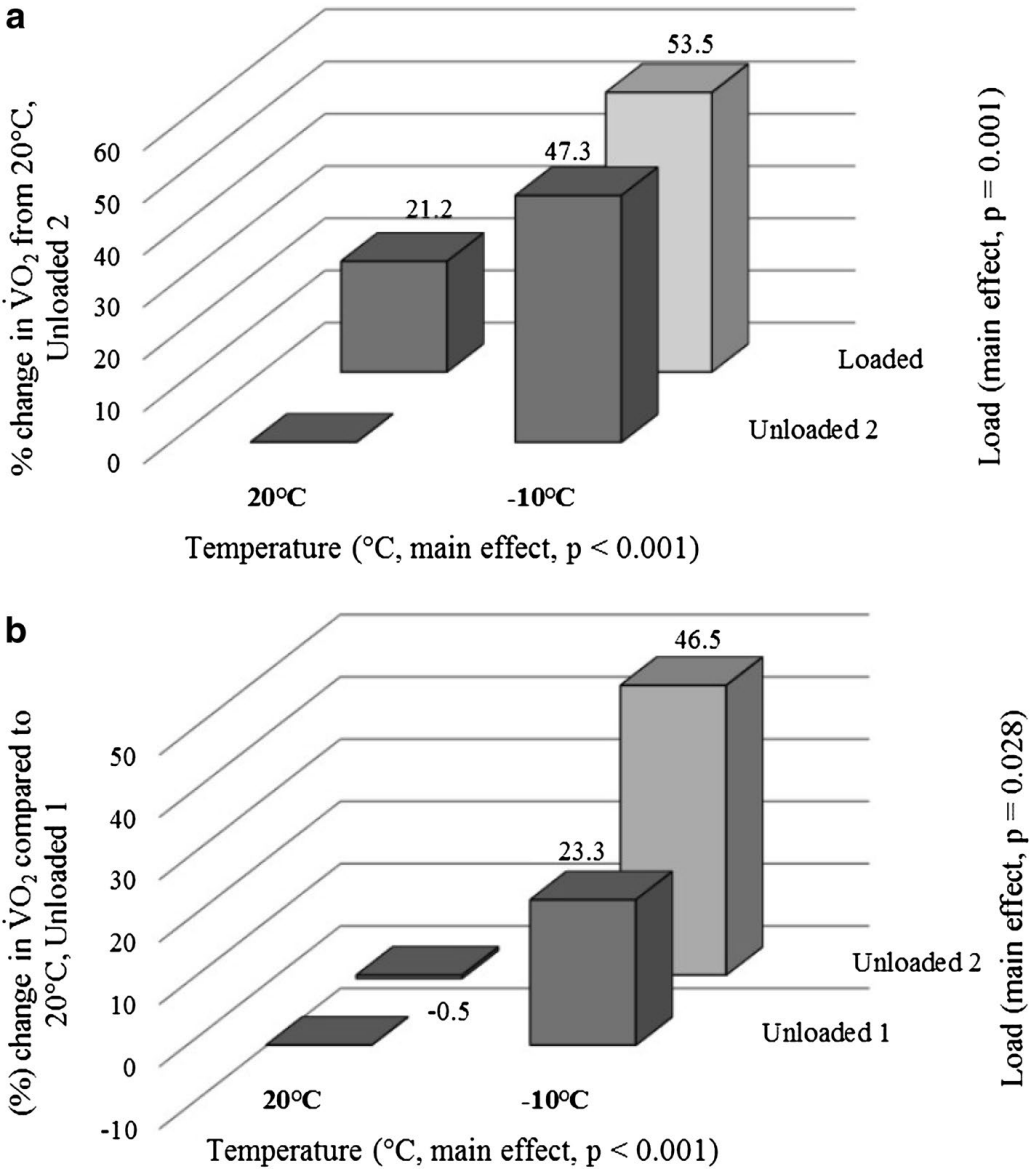
Interactions showing mean percentage change in V̇O_2_ (L · min^−1^) from 20 °C, unloaded 2 (**a**) and from 20 °C unloaded 1 (**b**) averaged across all gradients

Figure 3 shows the interactions between the temperature and the three load conditions. The pattern of response was similar between unloaded 1 and loaded walking. This was confirmed by no significant two-way or three-way interactions (*p* ≥ 0.127, *η*_p_^2^ ≤ 0.168). There was a significant interaction between temperature and load (*p* = 0.011, *η*_p_^2^ = 0.268) when comparing walking loaded with unloaded 2. At warmer temperatures, as expected, loaded and unloaded 2 exercise bouts produced different V̇O_2_ values, due to the effect of load. However, at colder ambient temperatures (−5 °C or −10 °C), V̇O_2_ values were similar for the second unloaded exercise and when carrying the 18.2 kg load. Figure 4a shows that a combination of decreasing temperature and increasing load produced an antagonistic (hypo-additive) interaction, including an increase of 47.3 ± 36.8% from 20 to −10 °C for unloaded 2, an increase of 21.2 ± 5.8% when backpack load was added and a combined increase of 53.5 ± 29.9% in V̇O_2_ at −10 °C for loaded compared to unloaded 2 at 20 °C. There was also a significant interaction between temperature and load (*p* < 0.001, *η*_p_^2^ = 0.392) when comparing unloaded 1 with unloaded 2 walking. The interaction is indicated by no differences at higher temperatures in contrast to the colder ambient temperatures (−5 and −10 °C) which produced higher V̇O_2_ values that were consistent with the loaded condition. At −10 °C, the mean difference between the two unloaded conditions was 0.29 ± 0.36 L · min^−1^ compared to −0.01 ± 0.09 L · min^−1^ at 20 °C. Figure 4b shows that a combination of low temperature and prolonged exposure (unloaded 2) produced a hyper-additive interaction. This comprised of an increase of 23.3 ± 18.1% from 20 to −10 °C for unloaded 1, a 0.5 ± 7.4% decrease from unloaded 1 to unloaded 2 at 20 °C, with an increase of 46.5 ± 37.4% in V̇O_2_ at −10 °C for unloaded 2 compared to unloaded 1 at 20 °C (mean difference = 0.59 L · min^−1^).

#### Heart rate

**Fig. 5:**
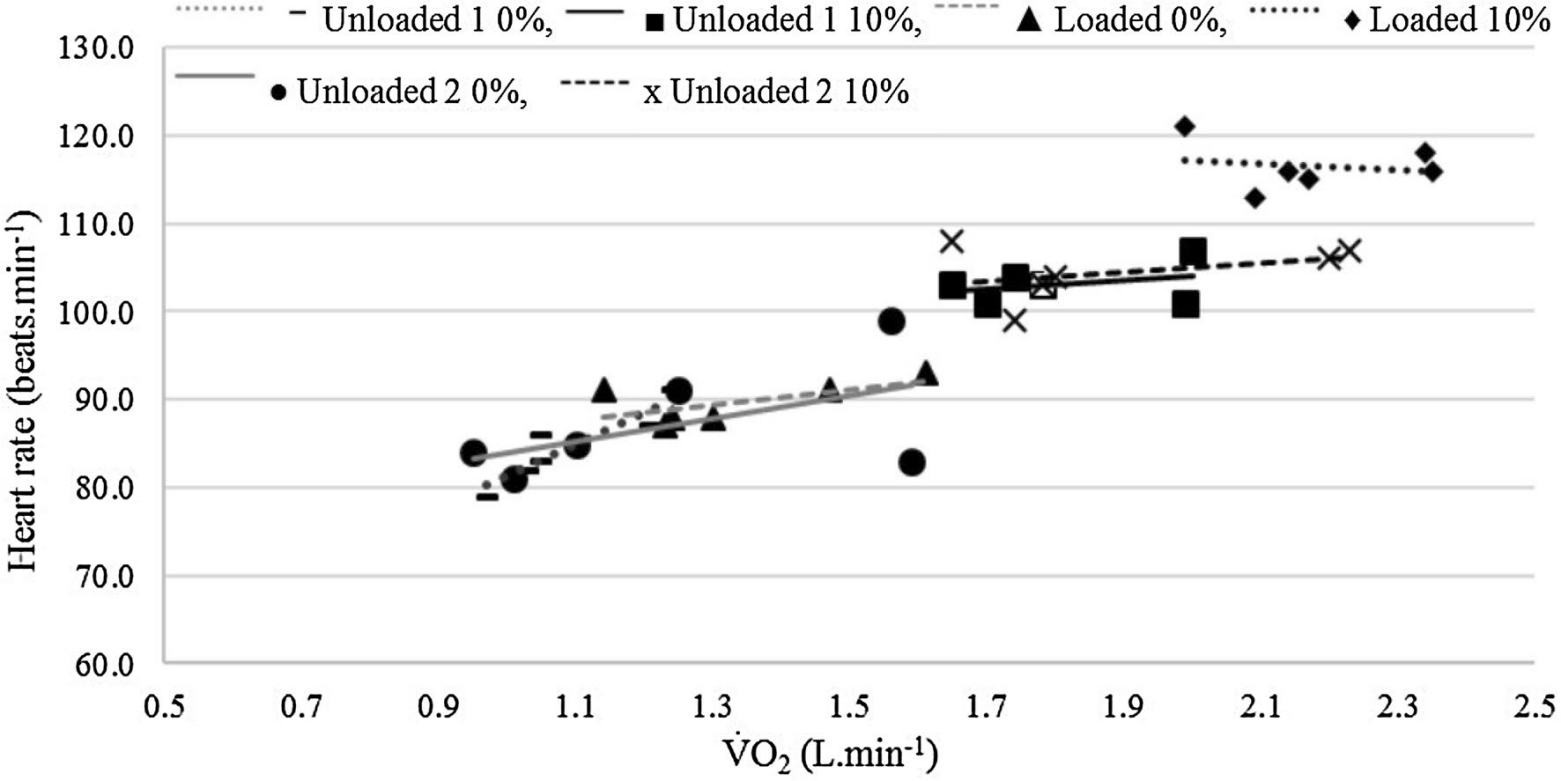
Correlation between absolute V̇O_2_ (L · min^−1^) and HR values (beats · min^−1^) for each phase with all temperatures included

HR values for some participants contained outliers, therefore, they were removed from the analysis, so* n* = 8. As for V̇O_2_, HR varied significantly across the three loaded conditions (*p* < 0.001, *η*_p_^2^ = 0.800) and was significantly greater for loaded walking compared to unloaded walking (*p* ≤ 0.001). HR was also significantly greater when walking uphill compared to level ground (*p* < 0.001, *η*_p_^2^ = 0.925). In contrast to V̇O_2_, ambient temperature did not significantly affect HR responses (*p* = 0.377, *η*_p_^2^ = 0.136, Fig. 1). In addition, when comparing unloaded 1 and unloaded 2, there were no significant differences in heart rate with temperature (*p* = 0.366, *η*_p_^2^ = 0.138). Figure 5 shows that across all conditions, the HR increased with V̇O_2_, as would be expected. However, within each of the conditions across the five different temperatures HR did not increase with V̇O_2_ in a systematic or consistent way as indicated by the line of best fit for each load and gradient condition in Fig. 5. For example, unloaded 1 0% shows a strong relationship between V̇O_2_ and HR (*r* = 0.91). In contrast, unloaded 2 at 10% shows a much weaker relationship (*r* =  0.37), accounting for only 14% of the variance. This is because the HR response at −5 and −10 °C is no different to the responses for higher temperatures, yet the V̇O_2_ increased by 1.0 L · min^−1^ from the warmest to the coldest temperature.

#### Peripheral and core temperatures

**Fig. 6:**
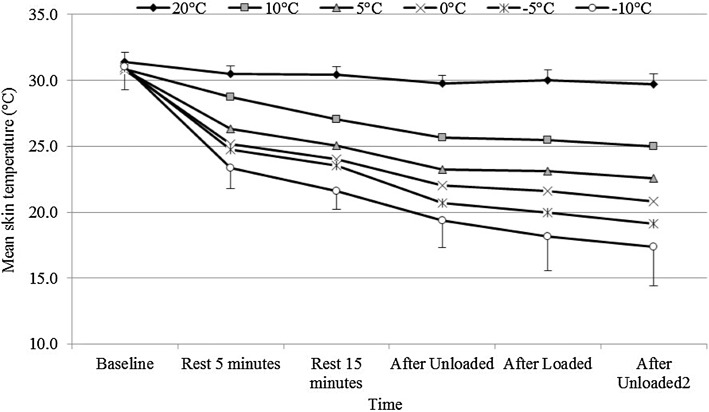
Mean ± SD Mean skin temperature (MST, °C) across the whole protocol for all temperatures

Skin temperature was recorded for 10 out of the 11 participants. Figure 6 shows that mean skin temperature (MST) was significantly reduced with decreasing temperature (*p* < 0.001, *η*_p_^2^ = 0.931). Similarly, there was a significant effect of time with baseline readings being significantly higher than those towards the end of exposure despite participants exercising (*p* < 0.001, *η*_p_^2^ = 0.966). Figure 6 also demonstrates an interaction effect for temperature and time (*p* < 0.001, *η*_p_^2^ = 0.748) showing a greater reduction in MST over time at colder ambient temperatures.

Core temperature decreased slowly throughout the −10 °C exposure by 0.75 ± 0.6 °C from a baseline value of 37.41 ± 0.45 °C.

### Kinematics

#### Effect of gradient

Walking uphill caused SL to be significantly greater than for level walking; 1.25 ± 0.08 m for 0% and 1.29 ± 0.10 m for 10% grade, demonstrated by a main effect of gradient (*p* = 0.008, *η*_p_^2^ = 0.564). Similarly, an increase in gradient also elicited a significantly greater trunk forward lean (TFL, ~4.43°) in all three gait phases (*p* < 0.001, *η*_p_^2^ ≥ 0.941).

#### Effect of load

There was no main effect of load across the three load conditions on SL (*p* = 0.871, *η*_p_^2^ = 0.015), however, backpack load caused TFL in all gait phases to be significantly greater regardless of temperature (*p* < 0.001, *η*_p_^2^ = 0.780).

#### Effect of temperature

As ambient temperature decreased, SL significantly decreased (*p* = 0.005, *η*_p_^2^ = 0.300). Combining gradients and loads, SL at 20 °C measured 1.29 ± 0.10 m compared to 1.25 ± 0.10 m at −10 °C. Post-hoc tests revealed a significant difference between 5 °C and −5 °C (mean difference = −0.024 m, *p* = 0.041). There were no significant interactions between temperature, with load or gradient (*p* ≥ 0.110) or a three-way interaction (*p* = 0.274, *η*_p_^2^ = 0.122).

There were mixed results regarding temperature and TFL. Mid-stance showed no significant differences (*p* = 0.115, *η*_p_^2^ = 0.174) between the different ambient temperatures. In contrast, measurements for left heel strike showed that participants leaned further forward at colder temperatures (a significant main effect for temperature: *p* = 0.029, *η*_p_^2^ = 0.235). Left toe-off showed a trend for greater TFL at colder temperatures (*p* = 0.054, *η*_p_^2^ = 0.209). Table 2 shows the main effect of temperature during left heel strike. There were no reported interactions for temperature with load or gradient with regard to TFL (*p* ≥ 0.299, *η*_p_^2^ ≤ 0.111).

**Table 2:** Mean ± SD Trunk forward lean (°) during left heel strike for all environmental temperatures averaged across all gradients and loads

Temperature (°C)	TFL (°)
20	94.7 ± 3.4*
10	94.9 ± 2.4
5	94.5 ± 3.5
0	94.6 ± 3.5*
−5	95.2 ± 2.4
−10	96.8 ± 2.8

*Denotes a significant difference to −10 °C (*p* < 0.05)

#### Interaction effects

A significant interaction for SL between load and gradient was reported when comparing unloaded 1 and loaded conditions (*p* = 0.001, *η*_p_^2^ = 0.656). The effect of gradient on SL was greater than that of backpack load. When a combination of both load and gradient occurred, SL was increased by 3.7 ± 4.3% above unloaded 0% walking. This was classified by Lloyd and Havenith (2016) as a hypo-additive (antagonistic) interaction, as gradient and loaded separately caused a 4.9 ± 3.7% and 1.4 ± 2.1% increase in SL, respectively. In contrast to V̇O_2_, when comparing the two unloaded conditions, prolonged exposure to lower ambient air temperatures had no effect on SL, as demonstrated by a non-significant interaction between temperature and load (*p* = 0.232, *η*_p_^2^ = 0.137).

**Fig. 7:**
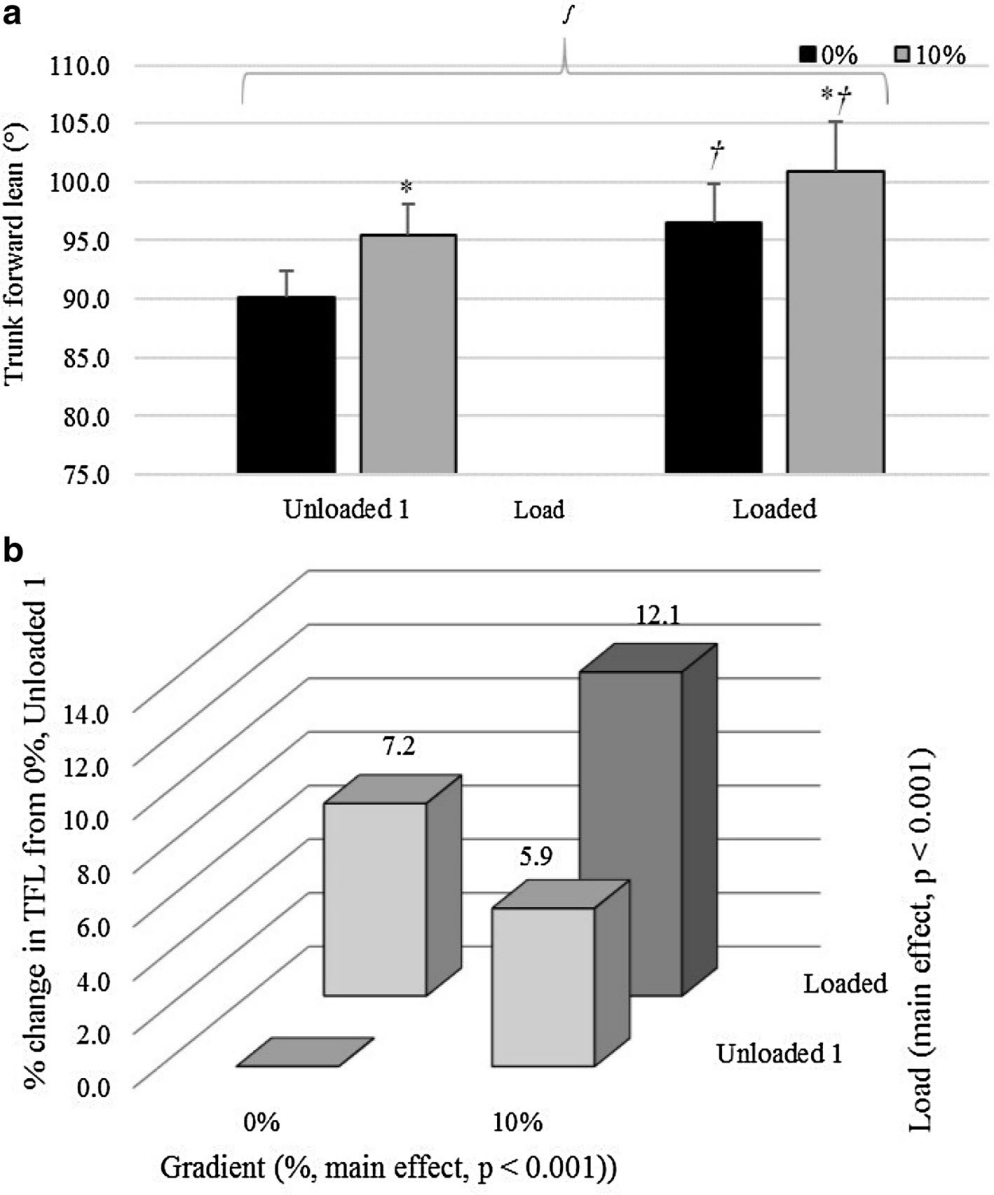
**a** Mean ± SD TFL (°) during toe-off for Unloaded 1 and Loaded at 0 and 10% gradient averaged across all ambient temperature. *Asterisk* denotes a significant difference to 0% responses for the same load, and *dagger* denotes a significant difference to unloaded responses for the same gradient. *Integral symbol* denotes an interaction effect for gradient × load. **b** Interaction showing mean percentage change in TFL (°) from 0% gradient, unloaded 1, averaged across all ambient temperatures

At left toe-off, when comparing unloaded 1 and loaded conditions, there was an interaction effect between load and gradient for TFL, as shown in Fig. 7a (*p* = 0.037, *η*_p_^2^ = 0.367). Figure 7a shows that backpack load had a greater effect on TFL than gradient. Figure 7b shows that the two conditions together (load and 10% incline combined) caused an additive relative effect (percentage increase, 7.2 ± 4.0% increase for load, 5.9 ± 1.7% increase for gradient, and 12.1 ± 4.7% increase when combined).

## Discussion

### Oxygen consumption

The first novel finding in this study was that the effect of load was not significantly different between the different ambient temperatures. Walking with an 18.2 kg backpack at −10 °C elicited a similar absolute ΔV̇O_2_ response to that at 20 °C for both level and uphill walking.

The increases in V̇O_2_ seen during both the two unloaded and loaded exercise bouts at sub-zero temperatures compared to thermo-neutral and cool temperatures indicate that the submaximal oxygen consumption was higher in the cold environment. The findings from this study add to the body of literature to show at what temperatures V̇O_2_ is significantly affected. Our results support the findings of Sandsund et al. (1998) who found that during cold exposure (−15 °C), V̇O_2_ was significantly higher (by 10.8%) during submaximal running exercise intensities. This is a smaller percentage increase compared to the present findings as the current study found on average, V̇O_2_ was 20.5% higher at −10 °C compared to 20 °C. Differences in the effect on V̇O_2_ could be due to the higher exercise intensities employed, as Sandsund et al. (1998) averaged V̇O_2_ over running intensities of 50–95%V̇O_2max_. Studies have shown that as exercise intensity increases, the effect of the cold becomes lessened due to an increase in heat production from the exercise (Pugh 1967). The results from the present study are also in agreement with findings by Oksa et al. (2004), who showed that at a workload of 25%V̇O_2max,_ V̇O_2_ was significantly higher during cold exposure (−15 °C) than at 20 °C (*p* < 0.05). Similarly, when exercising at 50%V̇O_2max,_ in 0 and −15 °C conditions_,_ V̇O_2_ significantly increased almost linearly when compared to results at 20 °C. Whilst V̇O_2_ was greater in the cold, reflecting findings by Oksa et al. (2004), the increase in V̇O_2_ was not linear. Results from the present study did differ to the previous findings as Oksa et al. (2004) reported that heart rate results mirrored those of V̇O_2_ at both intensities, whereas our findings showed that heart rate did not change in proportion to changes in V̇O_2_ with temperature. The participants in the study by Oksa et al. (2004) wore layered clothing weighing up to 4.9 kg at −15 °C. It is unclear how much of an effect this additional weight had on V̇O_2_, but it would be expected to increase it in absolute terms, although not relative to total mass, but this could explain the increased heart rate response found by Oksa et al. (2004).

The reasons for a higher V̇O_2_ response during cold exposure are likely due to reduced mechanical efficiency and thus increased co-activation of muscle pairs. Muscle strength has been previously demonstrated to be reduced during cold exposure and has been attributed to a number of factors including slowed calcium uptake, ATP utilisation and a reduction in cross-bridge force kinetics (Oksa et al. 2004). Wakabayashi et al. (2015) reported that during exercise in the cold, EMG amplitude has been shown to be increased. This evidence suggests that to maintain the same walking speed of 4 km · h^−1^, participants may have recruited more muscles fibres as the more superficial muscle fibres become weakened by the cold (Oksa 2002; Castellani and Tipton 2016). If more motor units are employed to meet the exercise demands this will increase V̇O_2_ as reflected in the present study by greater V̇O_2_ values at −10 and −5 °C.

Another likely cause for elevated V̇O_2_ associated with cold exposure is shivering in an attempt to maintain core temperature (Doubt 1991). The unparalleled activation of skeletal muscle fibres generates heat, increasing the requirement for carbohydrate oxidation which results in quicker glycogen depletion. The exercise intensity when walking with a load, however, was above the estimated 1.5 L · min^−1^ threshold for the shivering response; therefore, it is unlikely that this was the only explanation (Sandsund et al. 1998). In the present study and in agreement with Ito et al. (2013) and Castellani and Tipton (2016), the absence of overt shivering observed during exercise in some conditions combined with small change in deep body temperature and increased V̇O_2_ could be attributed to the generation of heat through non-shivering thermogenesis (NST). BAT releases norepinephrine which causes the breakdown of triglycerides, releasing fatty acids which in turn activates an uncoupling protein (UCP1) (Nedergaard et al. 2011). Substrate oxidation is then uncoupled or dissociated from the production of ATP, leading to an increased heat production (Cannon and Nedergaard 2004). Calorigenic hormones increase the oxygen consumption in most cells, increasing the basal metabolic rate, reflected as elevated V̇O_2_ during exposure to the cold (Ito et al. 2013). The contribution of NST to the whole body metabolic response is not fully understood, and Ouellet et al. (2012) reported a 1.8-fold increase in energy expenditure in participants after cold exposure. Total energy expenditure increased from 125.6 W (0.36 L · min^−1^) to 222.6 W (0.64 L · min^−1^), causing an average increase in V̇O_2_ of ~0.3 L · min^−1^. This was during an experiment conducted at a water temperature of 18 °C, and the contribution to energy expenditure during cold air temperatures should be considered in future research. Researchers, however, have concluded that BAT undoubtedly contributes to a cold-induced increase in energy expenditure and both NST and shivering contribute to the whole body metabolic response (Ouellet et al. 2012). The two mechanisms, thermogenesis and neuromuscular changes, do not work in isolation. Increased shivering and NST, particularly in the antagonist muscle, will also reduce mechanical efficiency. This occurs principally due to the increased requirement of the agonist muscle to produce a larger force/power output and thereby increased oxygen consumption to compensate for a higher antagonist/ joint resistance when the muscle becomes cold (Oksa 2002). The colder the ambient temperature, the greater the decrease in superficial muscle temperature meaning a greater reduction in muscle strength and blood flow, at least to superficial areas (Oksa et al. 2004). Vasoconstriction reduces blood flow to the muscle, and thus, oxygen delivery to working muscles may be reduced, leading to an earlier switch to anaerobic metabolism (Sandsund et al. 1998; Ito et al. 2013; Castellani and Tipton 2016). This combined with an increase in carbohydrate utilisation due to shivering would increase the accumulation of lactic acid, impairing performance and inducing fatigue (Jett et al. 2006). Muscle temperature was not measured in the present study; therefore, there is need for further work to evaluate this response.

The second novel finding from this investigation was the effect of prolonged exposure in the cold on V̇O_2_ as highlighted though the interaction between temperature and load. At sub-zero temperatures, the second bout of unloaded exercise elicited a significantly higher V̇O_2_ response to the first unloaded bout which was not observed when exercising at temperatures of 0 °C and above. This finding can be attributed to both mechanisms described previously, as the greater the time spent in the cold, the lower the core and skin temperature. This would elicit greater responses from defence mechanisms, such as shivering and additional recruitment of motor units to sustain workload and prevent hypothermia. This finding would support an increase in the %V̇O_2max_ that individuals would be working at when exercising in conditions of cold exposure which would induce faster fatigue and could lead to early termination of load carriage exercise. It could be suggested that the V̇O_2_ values shown when loaded at sub-zero temperatures could be affected by cold exposure too, as this bout of exercise occurs after unloaded 1 and the body would have had time to cool. However, the data in the present study show that this is very unlikely due to the effect of loading being stable across the temperatures, as shown in Fig. 3.

Gradient had a greater impact on V̇O_2_ than backpack load did. This can be attributed to the vertical work done during exercise on a gradient causing a greater increase in metabolism than extra load alone, as the body has to work to produce both horizontal movement and movement against gravity (Santee et al. 2001).

### Heart rate

There is no universally accepted response of HR to cold exposure. Within the literature, HR during submaximal exercise has been shown to decrease (e.g., Kruk et al. 1991; Spitz et al. 2014), increase (Oksa et al. 2004), or remain unchanged (e.g., Patton and Vogel 1984). Results from this study support the latter, as decreasing ambient temperature had no effect on HR responses. Vasoconstriction is likely to have occurred due to significant reductions in MST shown in the lower ambient temperatures; however, this had no effect on HR. It could be suggested that exposure time in the present study was not sufficient for the cold to influence HR. However, the duration of the protocol in the present study was 50–60 min, depending on the length of rest periods. Spitz et al. (2014) employed protocols of a similar duration (~60 min), and yet, they reported significant differences in HR responses. In addition, those authors did not use temperatures as cold as those in the present study. Consequently, protocol duration is unlikely to have been the sole reason for no change in HR. A difference in exercise intensity, exercise modality, or body composition could have influenced results.

### Biomechanical factors

Load reduces SL when walking on the horizontal and is regarded advantageous due to the decrease in stress on the bones of the foot (Knapik et al. 1996; Lloyd and Cooke 2011). However, most of the literature has reported only small decreases, and in the present study, no differences were found. In agreement with V̇O_2_ data, gradient caused a greater change in SL than backpack load. To counteract the resistance caused by gravity when walking uphill, individuals seek to increase momentum in their lower limbs which is achieved through increased SL (Leroux et al. 2002). TFL, however, displayed a different pattern of response with backpack load having a greater impact than gradient. This can be attributed to load affecting the CoM of an individual to a greater extent than gradient, causing participants to alter their posture to maintain stability (Singh and Koh 2009; Simpson et al. 2011).

Finally, the present study is able to add to the very limited evidence on changes in gait characteristics with ambient temperature. We found a reduction in SL to maintain the constant walking speed. Changes in spatiotemporal parameters could be attributed to the decrease in muscle tendon elasticity that occurs with decreasing temperature. Folland et al. (2006) proposed that stiffer tendons could reduce the amount of stored energy involved in the eccentric loading phase of running/walking which would reduce the net impulse distributed to the runner, decreasing stride length. For the runner or walker to sustain their speed, the solution would be to increase SF. Our results confirm findings by Folland et al. (2006) who showed that their cold trial caused significant increases in SF and concurrent decreases in SL when compared with gait parameters in thermo-neutral and hot conditions. They reported reductions of 0.04 m in SL in line with the reductions reported in the present study. Their cold exposure however was a pre-cooling exposure in water, and to date, there are no published investigations in cold air exposure on stride parameters, and this is the first study to report such findings.

A different mechanical response was demonstrated when walking uphill, which increased SL. This is in agreement with the few studies investigating stride parameters on varying gradients (Kawamura et al. 1991; Leroux et al. 2002; Fellin et al. 2016) yet in contrast to findings by McIntosh et al. (2006) who reported the opposite, with a decrease in SL as gradient increased from −10° to 0°. McIntosh et al. (2006) suggested the difference in their findings to other studies could be attributed to the length of the short walkway used to collect data (7 m) preventing participants from finding their normal gait pattern.

While temperature and gradient produced different responses in SL, both conditions produced significant increases in TFL. It has been suggested by Leroux et al. (2002) that as gradient increases, the momentum of the lower legs must also increase to overcome gravity, resulting in increases in SL. In this case, increased TFL is beneficial to the development of momentum in the lower limbs as moving body weight forward of the hip aids flexion of the hip. However, increased TFL can also be an adaptive response to decreased SL, particularly if muscle function is compromised (Li et al. 2013) as suggested in cold exposure conditions (Folland et al. 2006). Increasing TFL has the effect of moving the ground reaction force line anterior to the knee thereby aiding stability in the knee extensor-plantar flexion couple mechanism during limb loading (Kirtley 2006). The significant increases in TFL observed in the current study at left heel strike and left toe-off during cold exposure correspond with limb loading events in the gait cycle.

Previous studies reporting the effect of load on stride parameters are somewhat equivocal. Some authors have reported increases in SL with increasing load (e.g. Lloyd and Cooke 2011), whilst others report decreases in SL (Knapik et al. 1996; Harman et al. 2000). Knapik et al. (1996) proposed that a reduction in SL is advantageous due to the decrease in stress on the bones of the foot. The present findings associated with SL and load were similar to other studies, where no significant changes for SL were observed with increasing load (e.g., Singh and Koh 2009).

Phillips et al. (2016b) reported disproportional increases in V̇O_2_ with systematic increases in gradient when participants were walking at a constant speed. It was suggested that biomechanical changes, such as increased forward lean, were primarily responsible for these responses as TFL increased the muscle activity in the abdominals and lower back, which in turn required a greater V̇O_2_ (Phillips et al. 2016b). Biomechanical variables were not measured in the study by Phillips et al. (2016b); thus, the authors suggestions were purely speculative. The results from the present study show that although the oxygen cost of walking uphill with a load was disproportionately greater, the suggestion that this could be attributed to TFL alone can be refuted. The present study found that the change in TFL from the unloaded to loaded condition was greater during level walking than at a 10% gradient, whereas the effect of gradient upon V̇O_2_, was greater when unloaded compared with loaded walking. Biomechanical changes could indeed be responsible for the disproportional increases in V̇O_2_; however, the mechanism would appear to be more complex than simply increased TFL alone.

### Practical application

Understanding how load carriage affects physiological responses can provide information on how best to prepare for situations, where load carriage is a feature of the exercise challenge, such as in trekking or mountaineering. In terms of cold exposure, being inadequately dressed in cold environments would induce fatigue due to an increased V̇O_2_ response and a probable reduction in muscle strength with decreasing temperature for the same exercise intensity. Therefore, ensuring that skin temperature does not significantly decrease would be a major consideration when exercising in cold environments. When visiting cold climates, it is also worth bearing in mind the possibility of the extra energy expenditure required to undertake the same exercise challenge if clothing does not compensate for the effects of cold exposure. This has implications for training and fitness. Individuals should as much as possible employ task specificity to elicit the training response; therefore, they should adjust their training regimes to account for the extra energy requirement associated with exercising in cold ambient temperatures and/or with a load. Due to colder temperatures eliciting a greater V̇O_2_ response, individuals would be recommended to train at a higher intensity. At −10 °C, walking with a load required an extra average oxygen consumption of ~0.41 L · min^−1^ when compared to loaded walking at 20 °C and an extra ~0.67 L · min^−1^ when compared to unloaded walking at 20 °C.

### Limitations

Core temperature pills were only given for trials at −10 °C as a safety measure to ensure participants did become hypothermic. Ideally core temperature pills would have been used throughout; however, due to logistical reasons, this was not possible. For future work, measuring core temperature in all trials to see the effect of the protocol for a range of different ambient temperatures should be considered. In addition, no V̇O_2max_ data were collected for participants. The effect of cold exposure on V̇O_2max_ is well reported, but findings are inconsistent. Nonetheless, the majority of literature suggests that V̇O_2max_ does not change with a decrease in ambient temperature (Sandsund et al. 2012); therefore, as the effect of load did not change across the different ambient temperatures, it could be hypothesised that %V̇O_2max_ would be similar also. The same backpack was used for all participants; future work may consider using one model with various sizes, so that the pack can be custom fitted to each participant. The brief exercise bouts were also a limitation, but due to technical issues of freezing sample lines during cold exposure, exercise periods were kept to 14 min. As mentioned previously, Lloyd and Cooke (2000) and the current data showed that this time period was sufficient in establishing steady state.

## Conclusion

The evidence leads to a rejection of the first hypothesis as the results of this investigation show that ambient temperature had no significant effect on the increase in oxygen consumption associated with load carriage. The V̇O_2_ increase from unloaded to loaded walking for warm ambient temperatures is similar to that of very cold temperatures. However, the second hypothesis can be accepted, as a decrease in temperature elicited an increase in oxygen uptake. Prolonged exposure to the cold also saw V̇O_2_ significantly increase, with the second bout of unloaded exercise producing significantly higher V̇O_2_ in sub-zero temperatures than the first. The hypothesis regarding SL can also be rejected, as although increasing gradient elicited greater SL, increasing load and decreasing temperature did not interact with gradient to give a greater SL. SL decreased in colder temperatures, with load having no effect on SL.

Future research could focus on the magnitude and time course of the effect of prolonged exposure in the cold on increasing V̇O_2_ to establish if subsequent loaded periods would produce further significantly increases in V̇O_2_.

## Abbreviations

ANOVA: Analysis of variance
BAT: Brown adipose tissue
HR: Heart rate
MST: Mean skin temperature
NST: Non-shivering thermogenesis
V̇O_2_: Oxygen consumption
SL: Stride length
TFL: Trunk forward lean
